# Tumor board simulation improves interdisciplinary decision-making in medical students

**DOI:** 10.1007/s00432-024-05908-x

**Published:** 2024-08-30

**Authors:** Kevin Fink, Marie Forster, Matthias Oettle, Marcel Büttner, Chukwuka Eze, Lukas Käsmann, Amanda Tufman, Diego Kauffmann-Guerrero, Toki A. Bolt, Julia Kovacs, Jens Neumann, Johannes Mücke, Sonja Heuser, Stefanie Corradini, Franziska Walter, Maximilian Niyazi, Claus Belka, Martin Dreyling, Martin R. Fischer, Daniel F. Fleischmann

**Affiliations:** 1 Department of Radiation Oncology, LMU University Hospital, LMU Munich, Munich, Germany; 2 Institute of Medical Education, LMU University Hospital, LMU Munich, Munich, Germany; 3 Department of Medicine IV, LMU University Hospital, LMU Munich, Munich, Germany; 4 Department of Radiation Oncology, University of Tübingen, Tübingen, Germany; 5 Department of Medicine V, LMU University Hospital, LMU Munich, Munich, Germany; 6 German Center for Lung Research (Deutsches Zentrum für Lungenforschung, DZL), Comprehensive Pneumology Center Munich (CPC-M), Munich, Germany; 7 Division of Thoracic Surgery, LMU University Hospital Munich, LMU Munich, Munich, Germany; 8 Institute of Pathology, Faculty of Medicine, LMU Munich, Munich, Germany; 9 German Cancer Consortium (DKTK), Partner Site Munich, Munich, Germany; 10 German Cancer Consortium (DKTK), Partner Site Tübingen, Tübingen, Germany; 11 Bavarian Cancer Research Center (BZKF), Munich, Germany; 12 Department of Medicine III, LMU University Hospital, LMU Munich, Munich, Germany; 13 German Cancer Research Center (DKFZ), Heidelberg, Germany

**Keywords:** Tumor board simulation, Oncology, Decision-making, Medical education

## Abstract

**Introduction:**

Training of interdisciplinary clinical reasoning and decision-making skills, essential in daily clinical practice in oncological specialties, are still underrepresented in medical education. Therefore, at LMU University Hospital Munich, we implemented a didactically modified tumor board simulation with experts from five different disciplines (medical oncology, pathology, radiation oncology, radiology, and surgery) presenting patient cases into a one-week course on the basic principles of oncology. In this survey, we examined the self-assessed impact of our course on the interdisciplinary decision-making skills of medical students.

**Methods:**

Between November-December 2023 and January-February 2024, we surveyed two cohorts of medical students in the third year of medical school in our one-week course before and after participating in the tumor board simulation. The objective was to evaluate the self-assessed knowledge in interdisciplinary clinical decision-making, in integrating ethical considerations into clinical reasoning, and in comprehension of various professional viewpoints in interdisciplinary decision-making. Knowledge was assessed using a five-step Likert scale from 1 (no knowledge) to 5 (complete knowledge).

**Results:**

The survey was answered by 76 students before and 55 after the simulation, equaling 60–70% of all 100 course participants. Mean knowledge level regarding principles of interdisciplinary clinical decision-making improved significantly in all of the following exemplary aspects: purpose and procedure of tumor boards in clinical practice (from 2.4 ± 1.1 to 4.0 ± 1.0, Spearman’s ρ = 0.6, *p* < 0.001), principles of dealing with ethical challenges in oncology (from 2.4 ± 1.1 to 3.4 ± 1.0, ρ = 0.4, *p* < 0.001), and principles of shared decision-making in oncology (2.7 ± 1.1 to 3.7 ± 1.0, ρ = 0.4, *p* < 0.001). Students reported that their skills in clinical decision-making and ability to discuss oncological patient cases from different professional viewpoints improved due to the teaching course.

**Conclusion:**

By employing our interdisciplinary one-week course and a didactically modified tumor board simulation featuring experts from various oncological disciplines, medical students’ comprehension of interdisciplinary clinical decision-making in oncology improved significantly.

## Introduction

Oncology is a constantly growing and evolving field with an ever-increasing level of complexity (Soukup et al. 2018). As a result, oncology is shifting towards stronger specialization and more interdisciplinary collaboration (Mattes 2016). Understanding the guiding principles of interdisciplinary collaboration and clinical decision-making are essential features of the modern clinical oncological environment but they are still underrepresented in medical education (Kanan et al. 2022; Hall and Weaver 2001). Therefore, we implemented an interdisciplinary one-week course at LMU University Hospital about the basic principles of oncology, including a tumor board simulation at the beginning of the clinical phase of medical school. The main objective of this survey was to evaluate the effect of our interdisciplinary teaching course on the understanding of interdisciplinary clinical reasoning, i.e., interdisciplinary decision-making among medical students.

The tumor board simulation encompassed all essential aspects of a real-world tumor board, like the interdisciplinary discussion of oncological patient cases for developing a consensus therapy recommendation and encouraging evidence-based treatment options (Wright et al. 2007; Specchia et al. 2020). In this tumor board simulation, experts from five different fields (medical oncology, thoracic surgery, radiation oncology, pathology, and radiology) presented real patient cases in a didactically modified way, and students could decide upon therapy recommendations.

As ethical considerations are essential in oncological clinical practice (Doukas et al. 2015), e.g., ethical considerations in decision-making (Andersson et al. 2022), shared decision-making (Lawson McLean and Lawson McLean 2024), and dealing with therapy goal change or therapy complications (de Haes and Koedoot 2003), we examined the effect of our teaching course on these aspects.

In this survey, we examined our newly implemented interdisciplinary teaching format and assessed the effect on medical students’ comprehension of this clinical reasoning, i.e., the interdisciplinary decision-making process.

## Methods

### Survey setting and participants

The survey was conducted at the LMU University Hospital in Munch, Germany, with medical students in their first clinical semester, which is at the beginning of their third year overall. Part of the medical curriculum for these students is a one-week teaching course on the basic principles of oncology. Two weeks before the start of the course, we sent out an initial survey to students who were eligible to take part in the course. Following the course, we administered a second survey to evaluate the change in knowledge and comprehension.

### Questionnaire design and preparation

The survey questionnaire was prepared with evasys software (V9.1, evasys GmbH, Lüneburg, Germany), and the responses were collected and analyzed anonymously in accordance with the Medical Faculty of LMU Ethics Committee Guidelines.

The first questionnaire, before the teaching course, contained 45 items for self-evaluation, including one single-choice question, three open-ended questions, seven six-point-scale questions, and 34 five-point-scale questions. The second questionnaire, after the teaching course, included the same questions plus two single-choice questions and eight five-point-scale questions for self-evaluation of knowledge gain due to the teaching course. The single-choice questions were used to differentiate between students, open-ended questions were used for further evaluation and suggestions, and the five-point and six-point-scaled questions were used for self-evaluation of knowledge levels. On the five-point-scale questions, increasing values meant increasing levels of knowledge. On the six-point scale (used for self-evaluation of prior knowledge), analogous to the German school grading system, lower values indicated higher degrees of knowledge.

### Survey implementation

The survey was sent out to all students in their first clinical semester two weeks before the teaching course via email by the coordinators of the first clinical semester and was closed on the morning of the first day of the teaching course. The week before the course, students received a reminder email to take part in the survey. The survey was conducted in the winter semester 2023/2024. Due to higher student numbers in winter semesters, the curriculum is split into two halves, so the one-week teaching course was offered twice. After the course, students could fill out the survey either immediately via scanning a QR-code or by answering the survey by the invitation they received via email. We aimed to optimally survey all course participants to maximize the interpretability of the results by employing multiple invitations as described above.

### Project overview

The one-week block course on oncology principles was initially developed (Oettle et al. 2023) following the six-step approach to curricular development in medical education by Thomas et al. (Thomas et al. 2016). There, we determined the goals and objectives of the course to be the “consolidation of the students’ knowledge on the principles of oncology” as well as the “application of competency-based learning” (Oettle et al. 2023) as defined by the “National Competence-Based Learning Objectives Catalogue for Medicine” (NKLM) (Fischer et al. 2015).

The course currently consists of 18 sessions, ranging between 15 and 90 min. The sessions were held by 30 lecturers from 16 different disciplines, two more disciplines since our last publication (Oettle et al. 2023). To deepen the understanding of the diagnostic modalities in oncology, we added a session on clinical laboratory medicine, and the session on imaging in oncology is now interdisciplinary, with a nuclear medicine specialist in addition to the radiologist.

One of the core elements of our one-week teaching course is the interdisciplinary tumor board simulation, in which five experts (thoracic oncology, thoracic surgery, radiation oncology, pathology, and radiology) discuss several thoracic oncological patient cases in a didactically modified way and students can decide on the therapy recommendation. This aligns with the new edition of the “National Competence Based Learning Objectives Catalogue for Medicine”, NKLM 2.0, shifting from fact-based learning towards competency-based learning (Dapper et al. 2022). Our one-week teaching format, with the virtual tumor board, aims to teach students the principles of interdisciplinary decision-making and consider ethical aspects in clinical practice beyond just factual knowledge about oncological disciplines. We evaluated the impact of our teaching course on students’ knowledge levels using this two-part survey.

### Multidisciplinary tumor board simulation

The tumor board simulation was held in a lecture theater with a presentation slide showing key clinical features. Five expert lecturers (from thoracic oncology, thoracic surgery, radiation oncology, radiology, and pathology) were presenting the clinical case. Radiological and pathological findings were explained by the respective experts. Then, the therapeutic experts expressed their opinions about possible treatment options.

After all the expert opinions, students could decide on their recommendation for a treatment plan by choosing one of five options. Due to the high number of student participants, the voting was conducted majority-based. After the students’ voting, the clinical discussion entered a second stage, in which the experts decided upon their treatment recommendation and explained their reasoning behind the decision and why or how it differed from the students’ decision. They also discussed and explained why the other options were not viable in their view, or when two recommendations were similarly viable, explained their reasoning as well.

Importantly, students were able to engage with the expert recommendation immediately after deciding on a therapy recommendation.

Exemplary contents of the tumor board simulations were a UICC stage IIIB non-small-cell lung cancer where surgical resection was not feasible, so the tumor board consensus was radiochemotherapy. Here, students had to understand the surgical viewpoint, i.e. that surgical intervention was not feasible in this scenario. In another case, a patient was presented with extensive-stage small-cell lung cancer who was diagnosed with acute COVID-19 while receiving chemotherapy. In this case, students needed to understand the deliberation between the toxic effects of chemotherapy and the necessary treatments for acute infection.

At the end of each case, students received immediate feedback by the clinical experts on their clinical decisions, and remaining questions could be clarified. Additionally, ethical considerations were discussed, e.g., the deliberation of possible side effects as well as the deliberation of therapeutic goals, i.e. strongest possible prolongation of life vs. maintaining a high quality of life. Also, shared decision-making with the patient, as recommended by the tumor board, was emphasized in each case. That way, different nuances of patient cases were discussed in detail in the student-expert exchange.

### Statistical analysis

We collected the data using the evasys software (evasys GmbH, Lüneburg, Germany). Then, we prepared the data results with Microsoft Excel (Microsoft^®^ Excel^®^ 2019 MSO (Version 2401 Build 16.0.17231.20236) 64 Bit, Redmond, WA, USA) and statistically analyzed them with RStudio (version “2023.12.1 + 402”).

The five-point scale used for assessing the knowledge level was evaluated from 1 to 5 the following way: “very little knowledge” (1), “little knowledge” (2), “medium knowledge” (3), “rather much knowledge” (4), and “very much knowledge” (5). After the teaching course, students were asked to assess how much they agreed that their knowledge level improved due to the teaching course on a five-point scale. It was evaluated as: “totally disagree” (1), “disagree” (2), “neither agree nor disagree” (3), “agree” (4) and “totally agree” (5). For assessing prior contact with oncology, the five-point scale was evaluated from “no prior contact” (1) to “neither much nor little prior contact” (3) and “intensely involved” (5). In the six-point-scale, ratings were evaluated according to the German school grading system from 1 to 6, which are: “very good” (1), “good” (2), “satisfactory” (3), “sufficient” (lowest passing grade) (4), “poor” (5), and “insufficient” (6).

The items were grouped into several categories in the questionnaire and the analysis. Those were: “Prior Knowledge”, “Level of Knowledge in Oncology”, “Level of Knowledge in Interdisciplinary Oncology”, “Ethical Aspects in Oncology”, “Opinions and Suggestions towards the Oncological Curriculum LMU Munich”, and for the survey after the course “Self-Evaluation after the Course”. Due to non-normal distribution, the Wilcoxon test was performed to test for significant improvement. For each item on the questionnaire, we calculated the mean, standard deviation, median, and interquartile range (IQR) before and after the teaching course. Then, we performed the statistical analysis by testing for significance using the Wilcoxon test. Due to multiple testing, the significance level was Bonferroni-adjusted to α = 0.0023 (initial α / number of observed items = 0.05 / 22 = 0.0023). We calculated the Z-score, and as a measure of effect size, we calculated Spearman’s ρ (rho). Percentages were rounded to the nearest integer.

## Results

### Student characteristics and prior level of knowledge

The survey was answered 131 times, 76 before and 55 after the teaching course. This equals more than 70% before and more than 50% after the course of all approximately 100 course participants. Of the 55 students, who completed the questionnaire after the course, 46 (84% of the 55) also completed the questionnaire before participating in the teaching course. All students were in their fifth curricular semester, the first clinical semester. 8% of the participants had previously visited a real tumor board, and the mean prior contact with oncology on a five-point scale was 3 (± 1.0), which we evaluated as medium prior contact with oncology.

The prior oncological knowledge level was assessed on a six-point scale, analogous to the German school grading system from 1 (“very good”) to 6 (“unsatisfactory”). Surgery received the lowest rating (mean of 4.6 ± 1.3; median 5, IQR: 4–6), followed by Interdisciplinary Decision-Making (4.1 ± 1.5; median 4, IQR: 3–5), Ethical Aspects in Oncology (3.9 ± 1.5; median 4, IQR: 3–5), and Medical Oncology (3.8 ± 1.3; median 4, IQR: 3–5). Radiation Oncology (3.3 ± 1.1; median 3, IQR: 2.5-4), Radiology (3.0 ± 1.0; median 3, IQR: 2–4), and Pathology (2.8 ± 0.8; median 3, IQR: 2–3) received the highest ratings, obtaining a mean rating of 3 (“satisfactory”).

### Evaluation of the teaching course

The items were evaluated on a five-point scale from 1 (“very little knowledge”) to 5 (“very much knowledge”).

#### Basic oncological knowledge

First, we evaluated the Basic Oncological Knowledge Level (see Table 1): the items “Definition of Cancer and Oncology”, “TNM System”, and “Evidence-based Clinical Decision-Making” showed no significant improvement and just a small effect size ≤ 0.3, as the initial rating was high beforehand. Basic knowledge levels in “Diagnostic Modalities in Oncology” and “Therapeutic Modalities in Oncology” both improved significantly (*p* < 0.001) with a medium effect size of 0.4.

**Table 1:** Evaluation of students’ knowledge self-assessment

Knowledge level	Before course		After course		Wilcoxon test		
	Mean (St. Dev)	Median (IQR)	Mean (St. Dev)	Median (IQR)	Z	*p-value*	Spearman’s ρ
Basic Oncological Knowledge							
Definition of Cancer and Oncology	4.0 (1.0)	4 (3–5)	4.4 (1.0)	5 (4–5)	− 2.9	0.004	0.3
TNM System	4.0 (1.2)	4 (3–5)	4.3 (1.2)	5 (4–5)	− 1.9	0.061	0.2
Evidence-based Clinical Decision-Making	3.5 (1.1)	4 (3–4)	3.9 (1.0)	4 (3.5-5)	− 2.1	0.034	0.2
Diagnostic Modalities in Oncology	3.4 (0.9)	3 (3–4)	4.1 (1.0)	4 (4–5)	− 4.8	*p* < 0.001	0.4
Therapeutic Modalities in Oncology	3.3 (1.0)	3 (3–4)	4.0 (0.9)	4 (4–4)	− 4.0	*p* < 0.001	0.4
Interdisciplinary Oncology							
Purpose and Procedure of Tumor Boards	2.4 (1.1)	2 (2–3)	4.0 (0.9)	4 (4–5)	− 7.0	*p* < 0.001	0.6
Principles of Interdisciplinary Decision-Making	2.2 (1.0)	2 (1–3)	3.8 (0.9)	4 (3–4)	− 7.6	*p* < 0.001	0.7
Types of Tumor Boards in Clinical Practice	1.8 (1.0)	1 (1–2)	3.2 (1.2)	3 (2.5-4)	− 6.1	*p* < 0.001	0.5
Time of Clinical Management for Presentation at Tumor Board	1.8 (1.0)	2 (1–2)	3.7 (1.1)	4 (3-4.5)	− 7.7	*p* < 0.001	0.7
Relevant Disciplines in a Tumor Board	2.2 (1.1)	2 (1–3)	4.0 (0.9)	4 (4–4)	− 7.5	*p* < 0.001	0.6
Principles of Interdisciplinary Decision-Making from Expert Viewpoint:							
Surgery	1.8 (0.9)	2 (1–2)	3.7 (0.9)	4 (3–4)	− 8.1	*p* < 0.001	0.7
Medical Oncology	1.9 (1.0)	2 (1–2)	3.8 (0.9)	4 (4–4)	− 7.7	*p* < 0.001	0.7
Radiation Oncology	2.2 (1.1)	2 (1–3)	3.9 (0.9)	4 (4–4)	− 7.2	*p* < 0.001	0.6
Pathology	2.4 (1.1)	2 (2–3)	3.9 (0.9)	4 (4–4)	− 6.7	*p* < 0.001	0.6
Radiology	2.3 (1.1)	2 (1.5-3)	3.9 (0.9)	4 (4–4)	− 6.9	*p* < 0.001	0.6
Ethical Aspects and Considerations in Oncology							
Ethical Aspects in Oncology in General	2.6 (1.2)	2 (2-3.5)	3.3 (1.0)	4 (3–4)	− 3.8	*p* < 0.001	0.3
Ethical Principles in Clinical Decision-Making	2.4 (1.1)	2 (2–3)	3.4 (1.0)	4 (3–4)	− 5.0	*p* < 0.001	0.4
Dealing with Ethical Challenges	2.4 (1.1)	2 (2–3)	3.4 (1.0)	3 (3–4)	− 5.0	*p* < 0.001	0.4
Dealing with Therapy Rejection	2.4 (1.1)	2 (2–3)	3.2 (1.1)	3 (2–4)	− 3.6	*p* < 0.001	0.3
Shared Decision-Making	2.7 (1.1)	3 (2–3)	3.7 (1.0)	4 (3–4)	− 5.1	*p* < 0.001	0.4
Dealing with Therapy Complications	2.2 (1.0)	2 (1–3)	3.2 (1.1)	3 (2–4)	− 4.9	*p* < 0.001	0.4
Dealing with Therapy Goal Change	2.3 (1.1)	2 (1–3)	3.2 (1.0)	3 (2–4)	− 4.5	*p* < 0.001	0.4

A five-point scale was used to measure the level of knowledge (1 [very little knowledge] to 5 [very much knowledge]). Significance level Bonferroni-adjusted to α = 0.0023, p-value ≤ 0.001 (significant). Spearman’s ρ ≤ 0.3 (small effect size), > 0.3 and ≤ 0.5 (medium effect size, > 0.5 (large effect size)

#### Interdisciplinary oncology

Second, we examined the items about “Interdisciplinary Oncology” (see Table 1). Here, we found highly significant (*p* < 0.001) improvement in all areas and a large effect size > 0.5 with Spearman’s ρ ranging between 0.6 and 0.7, which we calculated as our measure of effect size. We divided the items into two subgroups, one about interdisciplinary aspects in oncology and tumor boards in general and the second subgroup about principles of interdisciplinary decision-making from different expert viewpoints.

**Fig. 1:**
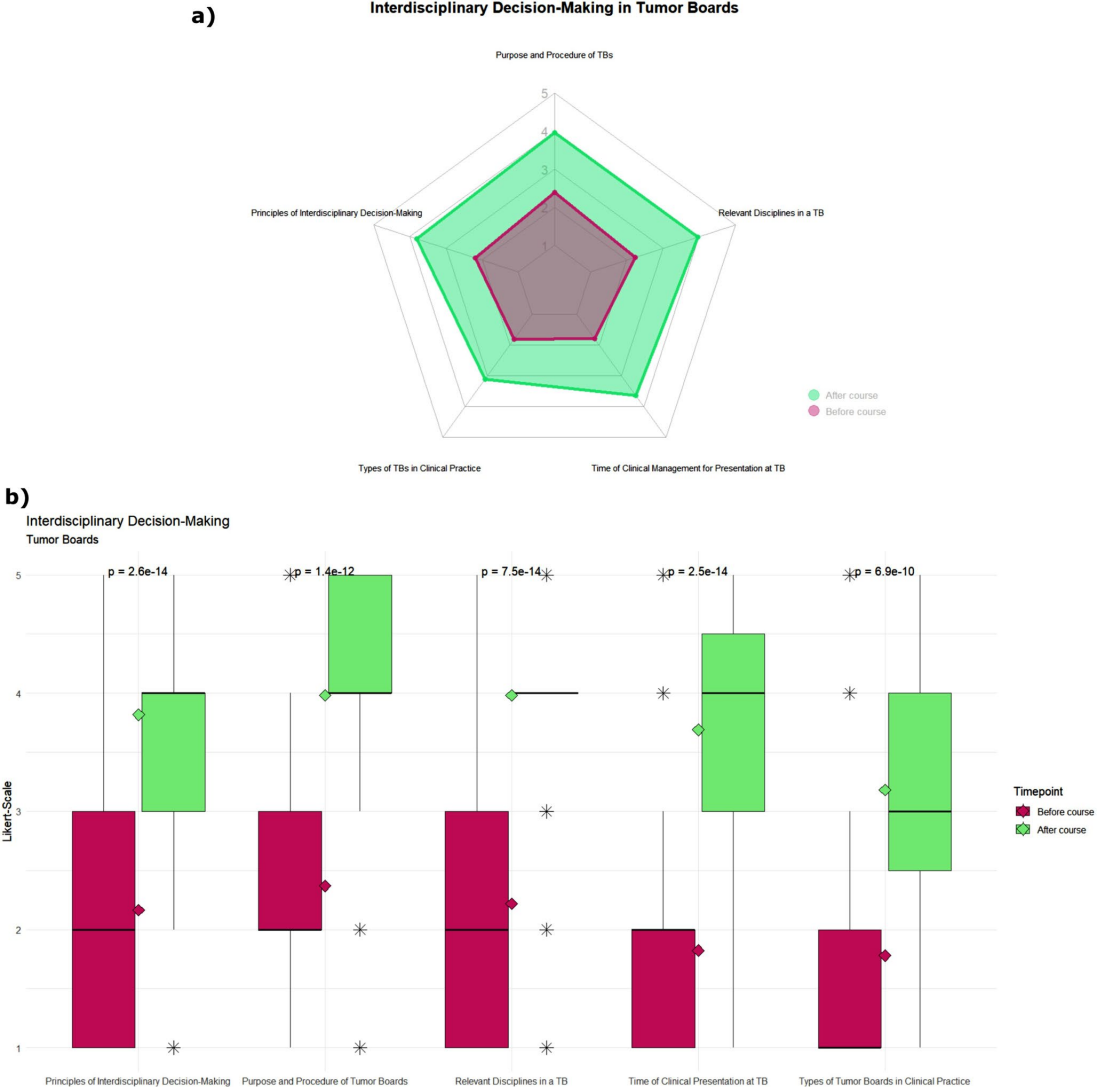
Impact of the teaching format on students’ self-assessed knowledge levels in the item group of interdisciplinary decision-making in tumor boards in general. Green: before course; red: after course. Knowledge level was assessed on a five-point scale from 1 [very little knowledge] to 5 [very much knowledge]. (**a**) Radar chart of the mean rating before and after the teaching course. (**b**) Boxplot of students’ responses, rhombus displays the respective mean rating, and the p-value was calculated using the Wilcoxon test

In the first subgroups (see Fig. 1), the items “Purpose and Procedure of Tumor Boards”, “Principles of Interdisciplinary Decision-Making”, “Time of Clinical Management for Presentation at Tumor Board”, and “Relevant Disciplines in a Tumor Board”, all improved from a median of 2 to 4. The fifth item in this subgroup, “Types of Tumor Boards in Clinical Practice”, improved from a median of 1 to 3. The largest improvement (ρ = 0.7) was found for the items “Principles of Interdisciplinary Decision-Making and “Time of Clinical Management for Presentation at Tumor Board” (see Table 1).

**Fig. 2:**
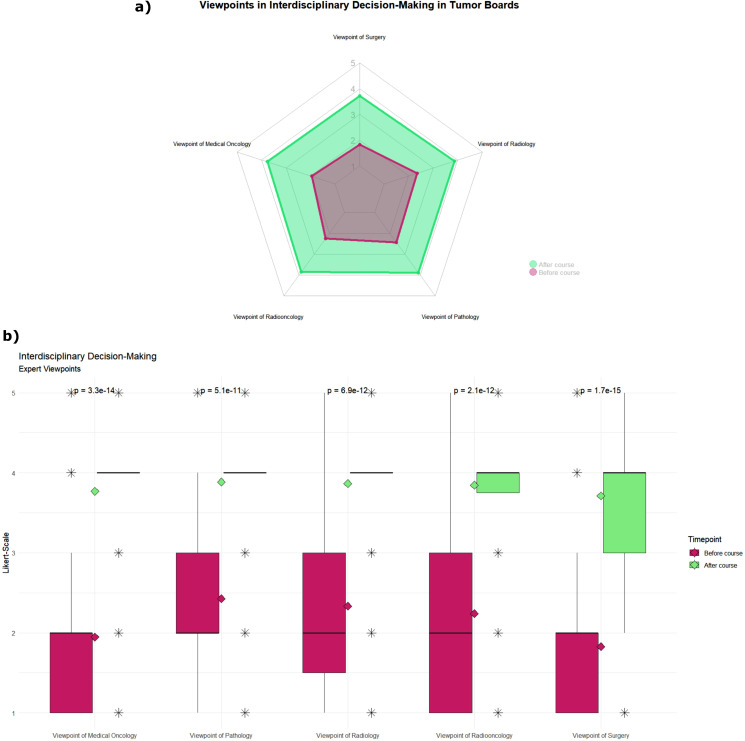
Impact of the teaching format on students’ self-assessed knowledge levels in the item group of expert viewpoints in interdisciplinary decision-making in tumor boards. Green: before course; red: after course. Knowledge level was assessed on a five-point scale from 1 [very little knowledge] to 5 [very much knowledge]. (**a**) Radar chart of the mean rating before and after the teaching course. (**b**) Boxplot of students’ responses, rhombus displays the respective mean rating, and the p-value was calculated using the Wilcoxon-test

In the second subgroup (see Fig. 2), all the items of the different expert viewpoints improved from a median rating of 2 to 4, the largest effect size Spearman’s ρ being “Surgery” (ρ = 0.7), followed by “Medical Oncology” (ρ = 0.7), “Radiation Oncology“ (ρ = 0.6), “Radiology” (ρ = 0.6) and “Pathology” (ρ = 0.6).

**Fig. 3:**
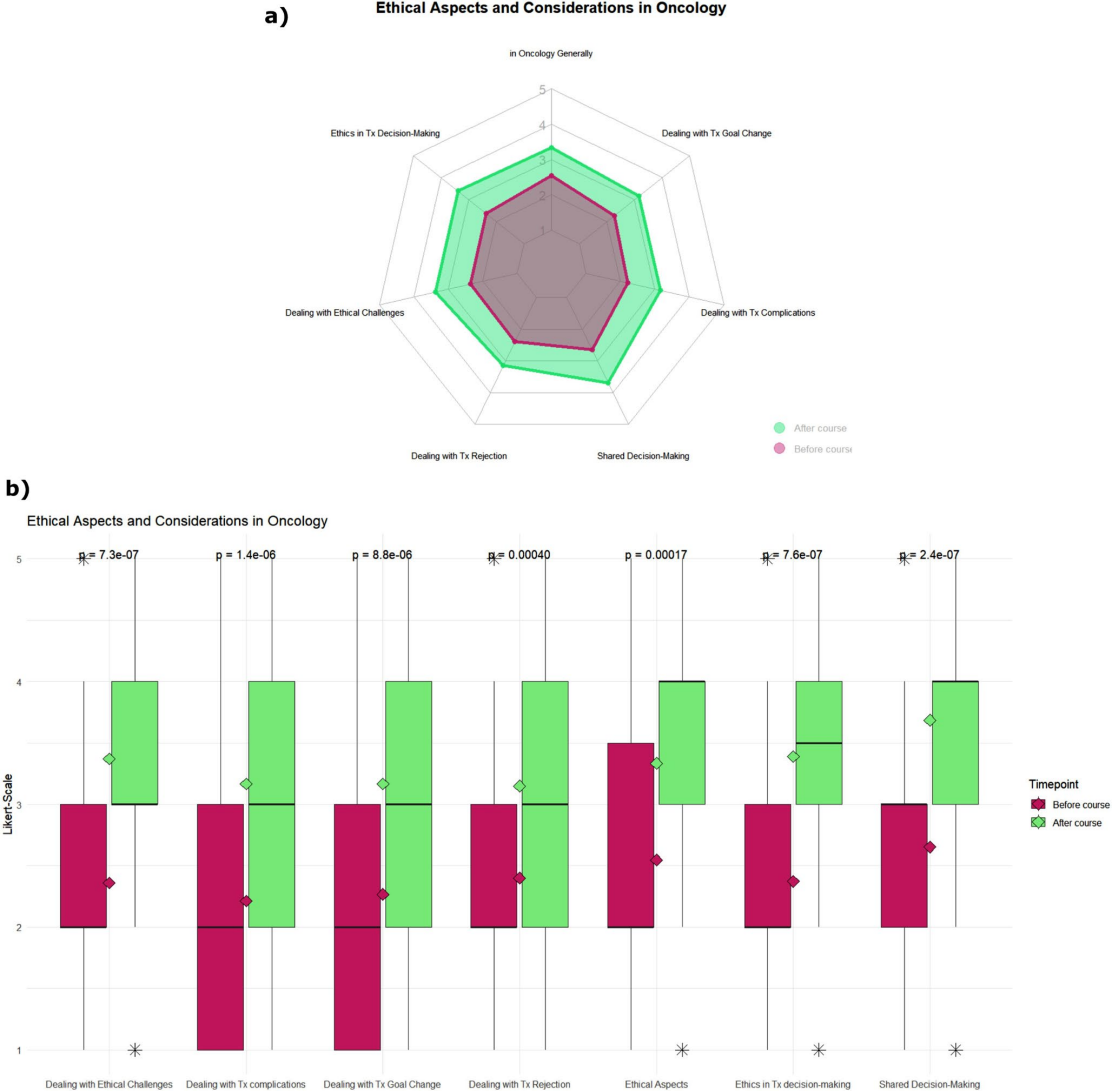
Impact of the teaching format on students’ self-assessed knowledge levels in the item group of ethical aspects and considerations in oncology. Green: before course; red: after course. Knowledge level was assessed on a five-point scale from 1 [very little knowledge] to 5 [very much knowledge]. (**a**) Radar chart of the mean rating before and after the teaching course. (**b**) Boxplot of students’ responses, rhombus displays the respective mean rating, and p-value was calculated using the Wilcoxon test

#### Ethical aspects and considerations in oncology

Third, we evaluated the item group “Ethical Aspects and Considerations in Oncology”. We found highly significant (*p* < 0.001) improvements in all observed items (see Table 1; see Fig. 3), just like in the second item group. The effect sizes here range in the medium effect size between 0.3 and 0.4. The median knowledge level before the course was “2” (“little knowledge”) in all items, except for “Shared Decision-Making” which received a median rating of “3” (“medium knowledge”). After the course, the median rating improved to ratings between 3 and 4 (4 being “much knowledge”), and the largest effect size was found in “Shared Decision-Making”, closely followed by “Ethical Principles in Clinical Decision-Making”, “Dealing with Ethical Challenges”, “Dealing with Therapy Complications” and “Dealing with Therapy Goal Change”. The items “Ethical Aspects in Oncology in General” and “Dealing with Therapy Rejection” improved with a borderline effect size between medium and low of 0.3.

#### Self-reported improvement after the teaching course

In the questionnaire after the teaching course, we included an item group for self-evaluation and self-reported improvement due to the course (see Table 2). Here, we used a five-point scale, which we evaluated from 1 (“totally disagree”) to 5 (“totally agree”).

**Table 2:** Self-reported improvement

Self-Reported Improvement due to the Course	Mean (SD)	Median (IQR)
Understanding the Principles of Interdisciplinary Decision-Making	4.2 (0.9)	4 (4–5)
Discussing Patient Cases from Different Discipline Perspectives	4.0 (0.8)	4 (4–4)
Incorporating Ethical Aspects into Clinical Practice	3.4 (1.1)	4 (3–4)
Incorporating Ethical Considerations into Clinical Decision-Making	3.5 (1.1)	4 (3–4)
Clinical Decision-Making Skills	3.8 (1.0)	4 (3–4)
Empathetic Patient Communication Skills	3.2 (1.0)	3 (3–4)
Clinical Management of Patients from Diagnosis to Therapy	4.0 (0.8)	4 (4–4)

To measure the self-reported improvement, we used a five-point scale, ranging from 1 (“totally disagree”) to 5 (“totally agree”)

We found that the median answer for all the knowledge- and comprehension-based items was 4 (“agree”), except for one skill-based item, “Empathetic Patient Communication Skills”, which was rated 3 (“neither agree nor disagree”). The highest mean rating was “Understanding the Principles of Interdisciplinary Decision-Making”, followed by “Discussing Patient Cases from Different Discipline Perspectives”, “Clinical Management of Patients from Diagnosis to Therapy”, and “Clinical Decision-Making Skills. Ethical aspects were rated slightly lower, with the highest self-reported improvement being “Incorporating Ethical Considerations into Clinical Decision-Making”, followed by “Incorporating Ethical Aspects into Clinical Practice”, and “Empathetic Patient Communication Skills”.

## Discussion

### The need for teaching of interdisciplinary principles in oncology

The increasing global demographic burden (Global Burden of Disease 2019 Cancer Collaboration et al. 2022; Ferlay et al. 2018) and the complexity of oncology require, first, a broader range of specialties and disciplines contributing to patient care (Soukup et al. 2018; Mateo et al. 2022) and, second, an increasingly important role of oncology in the future. Therapy recommendations are typically found by the consensus of experts in interdisciplinary tumor boards, and the treatment itself can only be carried out by the collaboration of several specialists. Advancing research in genomic profiling (Mateo et al. 2022) and precision oncology even shows the need for a wider expansion of necessary experts in molecular tumor boards, such as tumor geneticists and bioinformaticians (Rieke et al. 2018; Heinrich et al. 2023; Ma et al. 2024). In this fast-growing environment, it is essential for all medical professionals to understand, first, the guiding principles of oncology and, second, how interdisciplinary teams collaborate (Pershing and Fuchs 2013) and form collaborative decisions (Mäurer et al. 2023; Cooper et al. 2021; Gay et al. 2013). However, in medical education, different specialties still focus on their own field without teaching important interdisciplinary aspects (Mäurer et al. 2023), although multidisciplinary teaching approaches are effective in oncological education (Ha and Parakh 2018).

Current literature shows the importance of focusing on interdisciplinary aspects in oncology education (Vayani et al. 2023; Mäurer et al. 2023). Tumor boards have also been identified as an important educational platform (Mäurer et al. 2023; Vayani et al. 2023).

Furthermore, education in oncology lacks standardization even in residency training, as advocated by the European Society of Clinical Oncology and the American Society of Clinical Oncology (Dittrich et al. 2016), which is why oncological education should follow a comprehensive and evidence-based structure for all relevant disciplines, ideally beginning in medical education.

### Tumor board – from bedside to classroom

The key features of a tumor board, as described by Wright et al. (Wright et al. 2007), can be subdivided into the following components: involvement of a multidisciplinary team, patient-centered approach, case presentation, discussion and consensus decision-making, and treatment recommendations. All of those real-world tumor board components are represented in our tumor board simulation. The bureaucratic components, like having up-to-date technological equipment or having a tumor board coordinator (Wright et al. 2007), were excluded for didactic considerations.

Even before a tumor board can convene, it is necessary to know at which point of the clinical management patients should be presented at a tumor board. Students’ knowledge level on this item improved significantly after our course (see Table 1).

In our tumor board simulation, we represent experts from medical oncology, surgery, radiation oncology, pathology, and radiology – all described in the literature as essential specialties relevant to tumor boards (Wright et al. 2007; Specchia et al. 2020; Prades et al. 2015; Pillay et al. 2016). Students’ knowledge level regarding the relevant disciplines in a tumor board improved strongly after participating in our simulation (summarized in Table 1).

The patient-centered approach was the mode and structure throughout the whole simulation. Patient cases, including symptom presentation, history, and clinical examination, were presented by one of our clinical experts, and were then followed up by the diagnostic experts from radiology and pathology. Incorporating case-based learning approaches into medical education has been shown to enhance clinical reasoning and critical thinking (Ali et al. 2018; Berman et al. 2016; Hassoulas et al. 2017; Lee et al. 2013).

The main focus of our tumor board simulation relied on the components of discussion, consensus decision-making, and treatment recommendation. As we believe, those are the most intricate and complex areas of the clinical reasoning and decision-making process in oncology, but also, arguably, some of the most important ones. After the case presentation, the experts discuss the patient cases, usually beginning with the diagnostic disciplines of radiology and pathology and then followed by the clinical disciplines of medical oncology, surgery, and radiation oncology. In real-world tumor boards, these experts are supposed to add their expert knowledge and opinions to the cases (Wright et al. 2007), which is also realized in our simulation.

Beginning with the diagnostic workup, thorough diagnostic elaboration is essential to ensure appropriate treatment recommendations in oncology (Prades et al. 2015). In our tumor board simulation, the radiologist explains the relevant CT and PET CT scans and their significance for the patient. The pathologist proceeds with the relevant findings, e.g., PD-L1 status and its clinical significance for the treatment options.

Then, the discussion is followed by the clinical experts, who discuss their respective views on the patient’s case and what treatment options they could potentially provide for the individual patient. In our simulation, this represents the clinical discussion and decision-making process of the real-world tumor board, where all experts contribute to the individual case to find a consensus decision for a treatment recommendation (Wright et al. 2007). In our survey, we found substantial and highly significant improvement in students’ knowledge of the principles of interdisciplinary decision-making from each expert viewpoint (see Table 1; Fig. 2).

As described in the methods section, due to the structure of our tumor board simulation, students had the possibility to first express their own clinical opinion and then engage in a discussion with the clinical experts. Here, the expert discussion in exchange with the students was probably the most fruitful learning experience as students were confronted with their decisions and reasoning and could gain deeper insight into the decision-making process in a tumor board. Most likely, this change is reflected in the survey as the students’ knowledge on the items “Principles of Interdisciplinary Decision-Making”, and ”Purpose and Procedure of Tumor Boards” increased strongly and significantly (see Table 1).

At this second stage of discussion, even further critical aspects of the decision-making process were addressed, namely ethical aspects and considerations. In one case, for example, the patient had already received several cycles of chemotherapy but had not finished his treatment yet and then developed severe viral pneumonia during the height of the COVID-19-pandemic. There, students had to deliberate the risks of possible therapeutic interventions and could gain a deeper understanding of competing risks in therapeutic options.

### Limitations and scope of this study

One of the main limitations of our study is the limited number of participants in the teaching course and the limited number of teaching courses, as the course is only offered once to each student at the beginning of their third year. This limits the number of possible survey participants and, therefore, the statistical power. In order to maximize the response rate, we contacted each course participant multiple times before and after the course, and dedicated time for evaluation at the end of the course. We aimed at a response rate of at least 50% to minimize bias, which we exceeded with a 76% response rate before the course and a 55% response rate after the course. Although response rates for voluntary surveys such as ours have been shown to increase from an average of 53% to an average of 68% between 2010 and 2020 (Holtom et al. 2022; Wilson et al. 2024), the phenomenon of “survey fatigue” during the COVID-19 pandemic (De Koning et al. 2021) has led to a decline, which might still affect response rates today. Although we achieved our goal of a response rate of over 50%, complete participation in the evaluation would have been optimal to increase generalizability.

Furthermore, the study is limited by its non-longitudinal and single-institution design, which does not allow for extrapolation toward long-term knowledge retention and broad applicability across institutions and curriculum designs. As the teaching format is newly implemented, we aim to address this limitation in future work.

While the short duration of the teaching format can be easily replicated in other institutions, it remains to be examined whether it has long-term effects on medical students in later stages of their studies. We strongly encourage others to implement our teaching format and call for replication studies to validate our findings in different populations and institutions.

A longitudinal analysis of our teaching format will also allow us to examine the clinical translation of the level of knowledge and skills taught in our course. We aim to validate these effects in future work. In terms of future work, we plan to strengthen our institution’s interdisciplinary oncological teaching courses, further incorporating in-person student tumor board simulations and additional interdisciplinary teaching formats within the current oncology curriculum. By expanding into a comprehensive interdisciplinary curriculum, we will be able to mentor and follow our students through a greater portion of their medical education and, therefore, assess the long-term effects of the interventions described in our current work.

Furthermore, our study is limited by the self-assessment of knowledge, which is considered a subjective measure of knowledge (Sitzmann et al. 2010). While self-assessment is considered an important skill for medical professionals (Pisklakov 2014; Mann 2010), more objective measures such as objective structured clinical examinations (OSCEs) or practical exams could be used to validate the self-assessed data in future studies. In order to validate our results in future studies, we plan to develop an evaluation concept for standardized assessments of interdisciplinary decision-making processes, focusing on the most important aspects described here, such as the different professional viewpoints, the basic decision-making principles in tumor boards, and the consideration of the patient’s perspective.

While these factors limit the generalizability of our findings, our results indicate the effect of our novel teaching format. In this study, we examined the influence on the desired learning goals and were able to show the effectiveness of the course through self-assessment. Future studies need to address the objective and long-term effects and evaluate the translation of these learning outcomes into clinical application.

### Clinical decision-making including ethical considerations

In the tumor board simulation, clinical decision-making including ethical considerations were an essential part of the student-expert discussion. For example, when debating competing therapeutic risks and toxic side effects, the experts emphasized the need for patient involvement in the shared decision-making process. They emphasized that only the patients can decide to what degree therapeutic side effects and risks can be tolerated by them. Most probably due to those discussions, where experts explained their reasoning in concrete terms, and the students could be confronted with their own reasoning and ask remaining questions, items about ethical aspects and considerations in the oncological decision-making all improved significantly, e.g., “Ethical Aspects in Clinical Decision-Making”, “Dealing with Therapy Complications, or “Shared Decision-Making” (see Table 1, Fig. 3). In the open questions section of the survey (data not shown), students expressed their wish for more learning opportunities about ethical considerations in clinical decision-making. We are convinced that incorporating ethically challenging patient cases and scenarios into teaching formats in oncology, and openly discussing these complicated cases with students, enables students to better understand real-world oncology scenarios.

Patient-oriented approaches and early considerations of patient’s perspective and wishes, such as those in our student-teacher-discussion, are critical factors for future healthcare professionals to understand: on the one hand, they are key factors affecting whether or not interdisciplinary decisions and therapy recommendations are carried out (Blazeby et al. 2006; Mileshkin and Zalcberg 2006). On the other hand, greater satisfaction by team members with the therapy decision has been shown when both biomedical and ethical aspects are considered in the decision-making process (Lanceley et al. 2008). As the patient perspective is not prevalent in all clinical tumor board applications (Hahlweg et al. 2015), sensitization of students, being future doctors, towards this topic is essential. We achieved this by always considering the patient’s perspective in the expert discussions with the students.

In general, students highlighted the tumor board simulation as a highlight of the one-week course in the open questions section. Based on the results of this study, we are currently developing a new teaching format, in which the knowledge acquired in the current course can be consolidated further by placing students in the roles of tumor board physicians such as oncologists, surgeons and radiation oncologists in an in-person simulation.

## Conclusion

Tumor boards are usually part of later stages of medical curricula, and many medical students only experience them in late stages for the first time, if they do so at all. Early familiarization could help students better understand the complex clinical environment of oncological disciplines. The results of this survey show that an interdisciplinary tumor board simulation is suitable for medical students at an early stage of their clinical education and effectively teaches basic oncological principles and knowledge about interdisciplinary collaboration and decision-making in oncology.

## Data Availability

Data is provided within the manuscript.
